# Preoperative insomnia exacerbates postoperative sleep fragmentation in elderly arthroplasty patients: a prospective cohort study

**DOI:** 10.3389/fpsyt.2025.1720427

**Published:** 2025-12-10

**Authors:** Jiawen Su, Meng Kang, Xuan Lai, Qian Xiang, Yalin Wang, Yongzheng Han, Xiangyang Guo

**Affiliations:** 1 Department of Anesthesiology, Peking University Third Hospital, Beijing, China; 2 Department of Geriatrics, Peking University Third Hospital, Beijing, China; 3 Peking University Health Science Center, Beijing, China

**Keywords:** insomnia, arthroplasty, sleep fragmentation, sleep disturbances, microarousals, EEG-based sleep monitoring

## Abstract

**Background:**

Preoperative insomnia is prevalent among elderly arthroplasty patients, yet its impact on postoperative sleep disturbance (PSD) remains underexplored. This study investigated whether preexisting insomnia exacerbates postoperative sleep fragmentation in total hip/knee arthroplasty (THA/TKA) patients under spinal anesthesia.

**Methods:**

In this prospective cohort study 121 patients aged ≥65 undergoing THA/TKA were stratified by preoperative insomnia. Sleep was assessed via subjective metrics including Pittsburgh Sleep Quality Index (PSQI), Athens Insomnia Scale (AIS) and Insomnia Severity Index (ISI). Meanwhile, the EEG-based monitor and actigraphy were used to assess the objective sleep quality and conduct sleep staging.

**Results:**

Pre-existing insomnia exacerbates postoperative sleep fragmentation, manifested by persistently elevated microarousals (operative night to postoperation day2, POD2) and prolonged sleep latency. Selective REM suppression occurs in insomnia patients during the acute postoperative phase (operative night and POD1), independent of NREM duration alterations. Subjective sleep quality assessments revealed that the insomnia group demonstrated significantly higher AIS and ISI scores compared to the non-insomnia group.

**Conclusion:**

Preoperative insomnia independently exacerbates postoperative sleep fragmentation in elderly arthroplasty patients. The study was registered with the Chinese Clinical Trial Registry (Registration No. ChiCTR2400085184) on June 8, 2024.

## 1 Introduction

A normal sleep pattern and cycle is important to achieve normal function of physiological and mental processes (1–3). Stage N1 is light sleep and progresses to deeper sleep in stages N2 and N3. The deepest sleep stages are also called slow wave sleep (SWS) (4). Rapid eye movement (REM) sleep is the sleep stage where dreaming occurs. Normal sleep patterns show a marked rhythmicity with each cycle made up of stages N1, N2 and N3 and REM (5). Physiologically, sleep is thought to have a restorative function during REM and SWS phases, whereas lighter sleep stages promote hormone secretion facilitating protein synthesis and anabolic processes (6). Even though the specific role of sleep is yet to be thoroughly elucidated (7), there is little doubt that proper sleep is an important factor in rehabilitation after surgery (8–10).

Postoperative Sleep Disturbance (PSD) represents a prevalent surgical complication following total knee arthroplasty (TKA) or total hip arthroplasty (THA) (11, 12). Clinical manifestations of PSD include shortened total sleep time, early morning awakening, increased sleep fragmentation (13), diminished sleep quality, and frequent nightmares (14). Sleep fragmentation, a prevalent feature of sleep disturbances in the elderly, is primarily driven by a dysregulation of the brain’s arousal circuits, leading to perioperative adverse outcomes. A prior study of 105 patients reported that approximately 52% of THA patients and 57% of TKA patients described some disturbance of sleep in the early postoperative period (15). Objective sleep evaluations following TKA have demonstrated substantial decreases in sleep time, disruptions in sleep architecture, and near-elimination of rapid eye movement (REM) sleep predominating within the first 3 nights postoperatively (16). A growing body of evidence links sleep fragmentation and diminished sleep quality to an elevated risk of postoperative delirium, impaired cognitive function, and delayed physical recovery and functional mobility. Furthermore, the bidirectional relationship between sleep and pain suggests that poor sleep can lower pain thresholds, potentially complicating postoperative analgesia (17). Therefore, identifying patients at high risk for PSD is a crucial step towards mitigating these serious clinical complications and improving overall surgical recovery.

Contributions to PSD are likely multifactorial, with contributions from postoperative pain, opioid intake, stress, and anxiety related to surgery (18, 19). However, there is currently limited dedicated investigation into whether pre-existing insomnia comorbidity influences the development of postoperative sleep disturbances. Therefore, the aim of this prospective cohort study was to evaluate whether the preoperative insomnia exacerbate postoperative sleep disturbances, sleep fragmentation in elderly patients undergoing TKA or THA.

## 2 Materials and methods

### 2.1 Ethics and study design

This prospective cohort study was conducted at Peking University Third Hospital between June 2024 and May 2025. Ethical approval was obtained from the Medical Science Research Ethics Committee of Peking University Third Hospital (Approval No. IRB00006761-M2024317) on May 23, 2024. The study was registered with the Chinese Clinical Trial Registry (Registration No. ChiCTR2400085184) on June 8, 2024. Written informed consent was obtained from all participants or their legal representatives prior to enrollment.

### 2.2 Participants

Comprehensive information regarding the study protocol was provided to all participants before enrollment. Inclusion criteria were: (1) Age ≥65 years, without gender restriction; (2) American Society of Anesthesiologists (ASA) physical status classification I–III; (3) Scheduled for total hip arthroplasty (THA) or total knee arthroplasty (TKA) under spinal anesthesia. Exclusion criteria comprised: (1) History of neuropsychiatric disorders (e.g. delirium, Parkinson’s disease, Alzheimer’s disease); (2) History of intracranial surgery, cerebral hemorrhage, or cerebral infarction; (3) Alcohol dependence, or substance abuse; (4) Inability to communicate as assessed by investigators due to language barriers or hearing impairment. Participants were also excluded for: voluntary withdrawal, poor compliance, protocol violation, use of medications or methods affecting study outcomes, or loss to follow-up. The diagnosis of insomnia disorder was established in accordance with the International Classification of Sleep Disorders, Third Edition (ICSD-3) criteria (20).

### 2.3 Procedures

#### 2.3.1 Data collection

The following parameters were collected: age, gender, height, weight, educational level, ASA classification, perioperative visual analog scale (VAS) pain scores, Charlson Comorbidity Index (CCI), Activities of Daily Living (ADL), Frailty Scale (FRAIL), Patient Health Questionnaire-9 (PHQ-9) depression score, Generalized Anxiety Disorder-7 (GAD-7) anxiety score, Montreal Cognitive Assessment (MoCA), and Mini-Mental State Examination (MMSE). Additional data, including laboratory results, medical history, and comorbidities, were extracted from medical records.

Data collectors were qualified personnel who had completed standardized training and passed competency assessments. Demographic data were collected by two investigators in the hospital ward one day prior to surgery.

#### 2.3.2 Anesthesia and perioperative care

Standard monitoring included electrocardiography, non-invasive blood pressure, heart rate. All subjects received spinal anesthesia via L2–3 or L3–4 interspace puncture to minimize confounding variables. Following successful puncture, 10–15 mg of 0.5% hyperbaric bupivacaine was administered. Standard medications (ephedrine, urapidil hydrochloride, atropine, esmolol) were used to maintain blood pressure and heart rate within ±20% of baseline values. Postoperative analgesia was achieved with: 20 mL of 0.5% ropivacaine for femoral nerve block (TKA) or 30 mL of 0.3% ropivacaine for fascia iliaca compartment block (THA). Intravenous patient-controlled analgesia (PCA) with sufentanil (100 µg) plus metoclopramide (20 mg) diluted in 100 mL normal saline was administered for acute postoperative pain. The decision on whether to use PCA was based on the patient’s actual condition and personal preference.

#### 2.3.3 Sleep quality assessment

Sleep monitoring was conducted after the patient’s hospital admission. Participants were housed in standardized rooms (controlled light, temperature) devoid of noise, television, and computers. Nursing staff minimized noise during nocturnal care. Dietary restrictions excluded alcohol, nicotine, and caffeine-containing substances. Physical activity was limited throughout the observation period. Lights-off (20:00) and lights-on (06:00) times adhered to hospital policy.

The structured clinical interview was conducted by trained research personnel during the preoperative assessment based on ISCD-3. Subjective sleep quality (21) was assessed preoperatively using the Pittsburgh Sleep Quality Index (PSQI) (22). Insomnia symptoms and severity were evaluated preoperatively and for three consecutive postoperative days using the Athens Insomnia Scale (AIS) and Insomnia Severity Index (ISI). The research personnel responsible for collecting all postoperative subjective data were blinded to the patients’ group allocation throughout the study period. Objective sleep quality and architecture were monitored using sleep tracking wristband (Fitbit Charge; Fitbit, Inc., San Francisco, CA, USA) (23) and electroencephalogram (EEG)-based sleep monitors, which is a wearable, single-lead forehead sleep recorder, that transmits EEG signals to a smartphone or tablet for analysis, designed for clinical and home sleep monitoring (24).

#### 2.3.4 Sample size calculation

The primary outcome of this study was postoperative sleep fragmentation, quantified by the microarousals. Sample size estimation was based on preliminary experimental data showing microarousals of 27.8 ± 12.5 postoperatively versus 21.6 ± 8.9 preoperatively in insomnia patients. With a power of 0.80 and two-sided significance level of 5%, a minimum of 32 insomnia patients was required to detect this effect size. Accounting for a 20% dropout rate, the target enrollment for the insomnia group was set at 40 patients. Given the reported 35.9% prevalence of preoperative insomnia in elderly surgical populations, the total minimum sample size required was estimated as 112 participants.

### 2.4 Statistical analysis

Data analysis was performed using IBM SPSS Statistics software (Version 26.0). Continuous variables underwent normality testing (Kolmogorov-Smirnov test). Normally distributed data are presented as mean ± standard deviation (mean ± SD) and compared using independent samples t-tests. Non-normally distributed data are presented as median (interquartile range) and compared using Mann-Whitney U tests. Categorical variables were analyzed using Chi-square tests; Fisher’s exact test was employed for small sample sizes or expected frequencies <5.

## 3 Results

### 3.1 Demographic and clinical data

**Figure 1:**
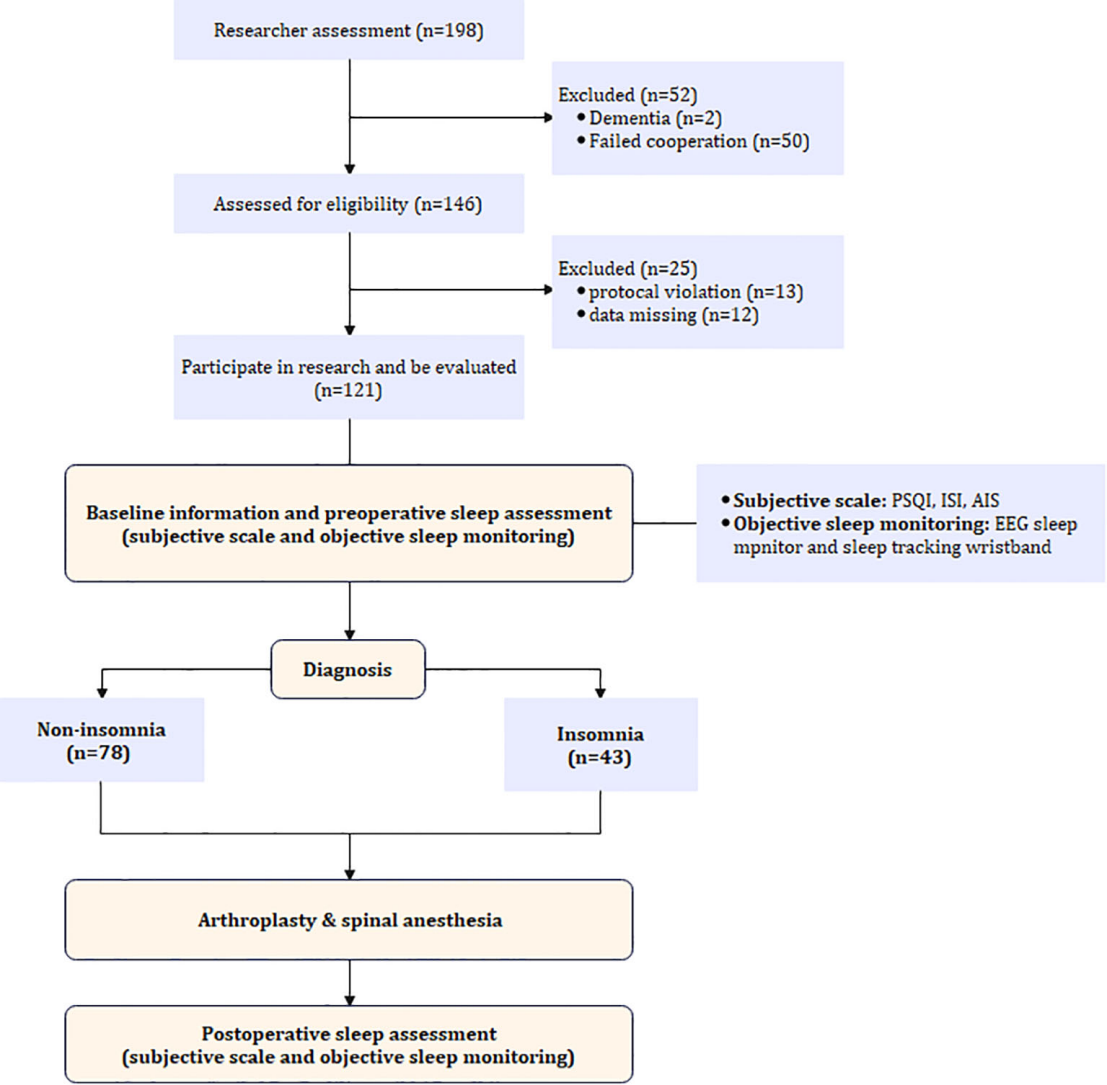
Flowchart of the study.

From June 3, 2024 to May, 2025, a total of 198 patients were screened for eligibility. Among them, 146 were eligible. During the study period, 12 patients had data missing, and 13 patients had protocol deviations. Consequently, 121 patients were ultimately analyzed and divided into two groups based on the presence or absence of insomnia before surgery, with 43 in the insomnia group and 78 in the non-insomnia group. (**Figure 1**).

In the study, preoperative insomnia was identified in 35.5% (43 in 121 participants) of enrolled patients. Baseline characteristics between the two groups—including age, educational attainment, ASA classification, preoperative pain scores, CCI scores, ADL scores, frailty and preoperative cognitive function—demonstrated overall balance. However, the insomnia group exhibited a significantly higher proportion of female participants (74.4%) compared to the non-insomnia group (*P* = 0.004). Additionally, patients with preoperative insomnia showed significantly elevated preoperative depression (*P*<0.001) and anxiety scores (*P* = 0.034) (**Table 1**).

**Table 1:** Baseline characteristics of included patients.

Characteristics	Non-insonmia group(n=78)	Insonmia group(n=43)	Statistical test	*P* value
Sex, No. (%)			-1.798	**0.004**
Female	47 (60.3)	32 (74.4)		
Male	31 (39.7)	11 (25.6)		
Age, years	71.4 ± 4.3	71.8 ± 4.2	-0.422	0.730
Education, years	9 (6)	9 (6)	0.262	0.794
ASA rating, No. (%)			2.060	0.357
I	12 (15.8)	5 (12.2)		
II	59 (77.6)	31 (75.6)		
III	5 (6.6)	5 (12.2)		
VAS				
Rest	1 (1)	1 (2)	-1.726	0.084
Activity	6 (2)	6 (2)	-0.231	0.817
CCI	0 (1)	1 (2)	-2.039	0.061
ADL	100 (2.5)	100 (0)	0.123	0.902
Frail Scale	0 (1)	1 (1)	-1.657	0.315
PHQ-9 depression score	0 (1)	2 (1)	-5.314	**<0.001**
GAD-7 anxiety score	0 (1)	0 (2)	-2.114	**0.034**
MoCA	23.2 ± 3.7	22.7 ± 3.9	0.482	0.577
MMSE	26.3 ± 2.8	26.1 ± 3.1	0.579	0.564

ASA, American Society of Anesthesiologist; ADL, Activity of Daily Living Scale; CCI, CharIson comorbidities index; VAS, Visual Analogue Scale; PHQ-9, Patient Health Questionnaire-9; GAD-7, Generalized Anxiety Disorder-7; normal distribution are expressed as mean ± (SD), SD, standard deviation; skewness distribution are expressed as median (IQR), IQR, interquartile.

Bold values indicate statistically significant differences, where the p-values are less than 0.05.

All patients received a standardized anesthetic and surgical protocol. No statistically significant differences were observed between the two groups regarding anesthesia duration, surgical duration, volume of infusion, volume of blood loss and postoperative patient-controlled analgesia (PCA) usage (all *P >*0.05) (**Table 2**).

**Table 2:** Surgical and anesthesia characteristics.

Surgical and anesthesia characteristics	Non-insonmia group(n=78)	Insonmia group(n=43)	Statistical test	*P* value
Duration of anesthesia, min	145.3 ± 25.4	143.6 ± 21.9	0.332	0.679
Duration of surgery, min	77.1 ± 22.9	78.7 ± 19.8	-0.421	0.589
Estimated volume of infusion, mL	1242.4 ± 137.0	1208.3 ± 145.5	0.222	0.202
Crystalloid solution, mL	803.0 ± 80.6	788.9 ± 90.9	0.220	0.825
Colloid solution, mL	439.4 ± 65.7	444.4 ± 61.7	-0.105	0.542
Estimated volume of blood loss, mL	22.8 ± 9.4	19.4 ± 7.8	0.508	0.130
Postoperative patient-controlled analgesia, No.(%)			0.176	0.675
Yes	16 (20.5%)	8 (18.6%)		
No	62 (79.5%)	35 (81.4%)		

### 3.2 Assessment of preoperative sleep quality

All enrolled patients underwent standardized sleep assessment on the preoperative day. Regarding subjective scale assessment, the insomnia group demonstrated significantly higher scores than the non-insomnia group on the Pittsburgh Sleep Quality Index (PSQI, *P* < 0.001), Athens Insomnia Scale (AIS, *P* < 0.001), and Insomnia Severity Index (ISI, *P* < 0.001). Objective assessment via EEG sleep monitor during the preoperative night revealed that insomnia patients exhibited reduced total sleep time (TST, *P* = 0.043), decreased sleep efficiency (SE, *P* = 0.036), prolonged sleep latency (SL, *P* = 0.039) and increased microarousals (*P* = 0.011). After adjusting for confounding factors, the results still showed significant differences (**Table 3**).

**Table 3:** Preoperative sleep assessment.

Preoperative sleep assessment	Non-insonmia group(n=78)	Insonmia group(n=43)	Statistical test	*P* value
Subjective scale				
PSQI	2 (2)	8 (3.5)	-7.373	**<0.001**
AIS	1 (2)	7.5 (2.5)	-5.342	**<0.001**
ISI	1 (1)	8 (3.5)	-5.172	**<0.001**
EEG sleep monitor				
Total sleep time (TST), min	364.6 ± 30.5	279.3 ± 69.3	2.075	**0.043**
Sleep efficiency, %	60.8 ± 14.8	45.8 ± 23.3	1.274	**0.036**
Sleep latency, min	51.5 ± 12.4	78.4 ± 24.4	-0.703	**0.039**
REM sleep duration, min	49.0(36.5)	40.1(36.7)	0.404	0.694
NREM sleep duration, min	256.2(101.2)	198.4(77.3)	0.938	0.368
Wakefulness after sleep onset (WASO), min	122.3(61.9)	192.2(74.3)	-1.152	0.274
Slow wave sleep (SWS) duration, min	15.8(6.7)	18.3(9.5)	-0.521	0.614
Microarousals	11.5 ± 4.5	22.5 ± 10.0	-2.522	**0.011**
Sleep tracking wristband				
Total sleep time (TST), min	355.2 ± 50.2	268.3 ± 55.0	1.517	0.172
REM sleep duration, min	44.0(22.5)	42.0(19.0)	1.227	0.292
Light sleep duration, min	250.0(105.5)	235.0(111.0)	0.891	0.566
Deep sleep duration, min	41.0(28.5)	35.0(30.5)	0.568	0.193

Bold values indicate statistically significant differences, where the p-values are less than 0.05.

However, sleep tracking wristband data showed no significant difference in TST (*P* = 0.172) between the two groups, which diverged from EEG sleep monitor findings. Furthermore, no statistically significant differences were observed between groups in REM sleep duration, light sleep duration, and deep sleep duration (all *P* > 0.05) (**Table 3**).

### 3.3 Postoperative sleep quality assessment

#### 3.3.1 Subjective scale

**Figure 2:**
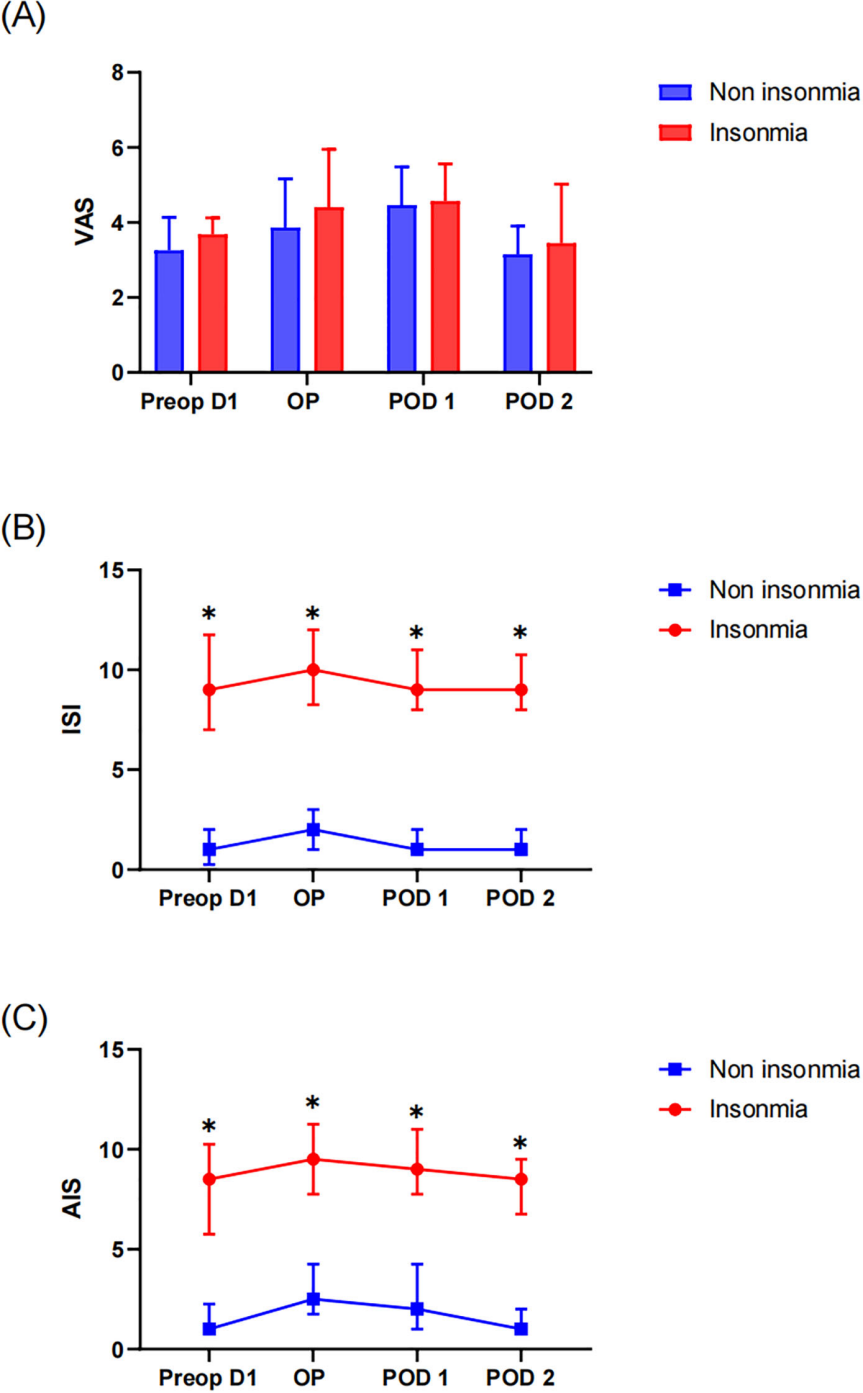
Perioperative subjective scale assessment: **(A)** VAS of pain, **(B)** Insomnia Severity Index (ISI) and **(C)** Athens Insomnia Scale (AIS). * indicates a significant difference between the insomnia group and the non-insomnia group.

Patients underwent pain assessment for three consecutive postoperative days. No significant differences in VAS pain scores were observed between the insomnia and non-insomnia groups during this period (**Figure 2a**). Subjective sleep quality assessments revealed that the insomnia group demonstrated significantly higher AIS and ISI scores both preoperatively and on each postoperative day compared to the non-insomnia group (all *P* < 0.05). When compared to preoperative baseline measurements, neither group exhibited statistically significant changes in AIS or ISI scores (**Figures 2b, c**).

#### 3.3.2 Objective sleep monitoring

**Figure 3:**
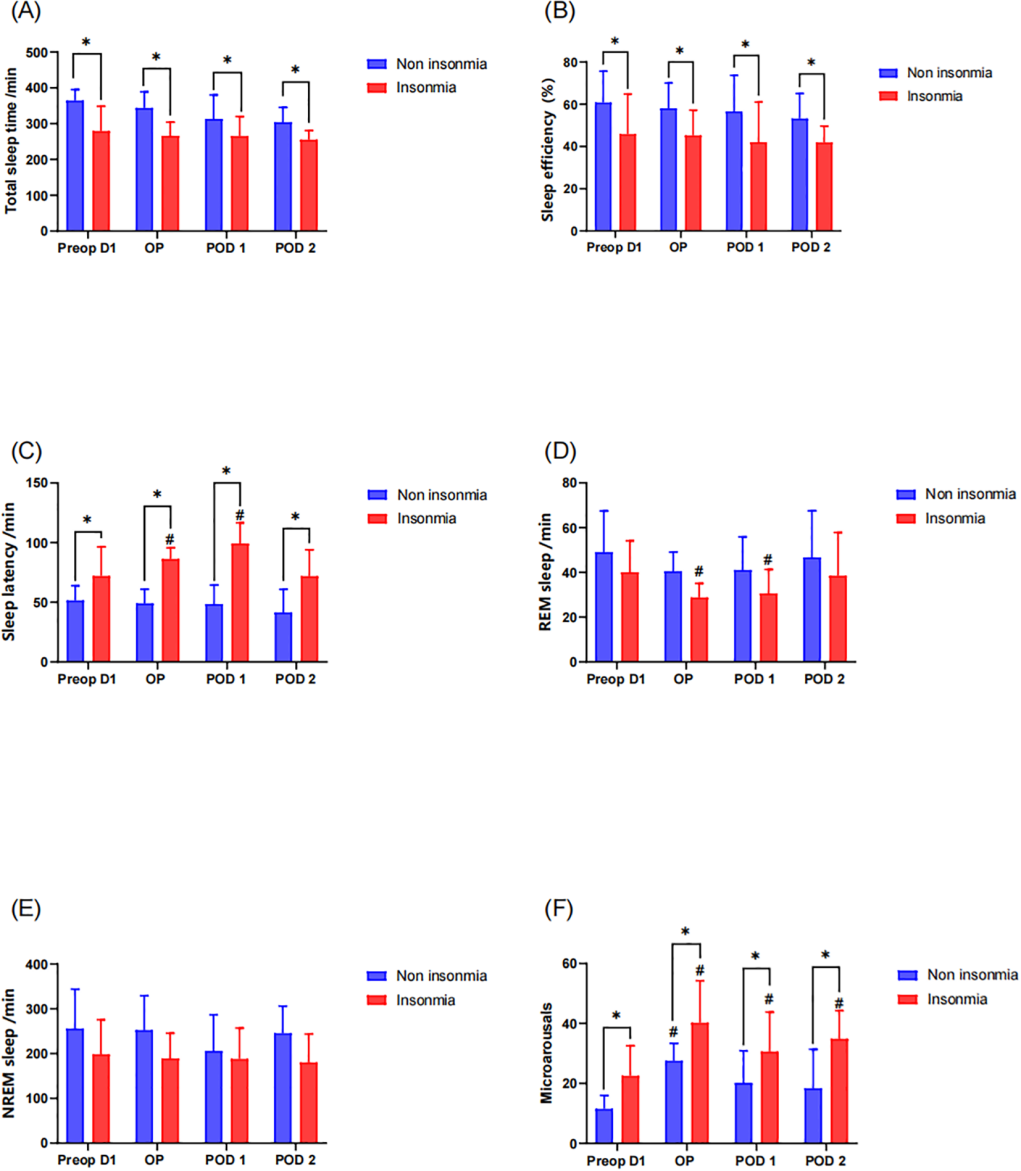
Postoperative sleep assessment by the EEG sleep monitor: * indicates a significant difference between the insomnia group and the non-insomnia group, while the # represents a significant difference compared to pre-operation D1. **(A)** Total sleep time; **(B)** sleep efficiency; **(C)** sleep latency; **(D)** REM sleep; **(E)** NREM sleep; **(F)** microarousals.

Objective sleep parameters were recorded via EEG sleep monitor from the operative night through the postoperative day 2. Results demonstrated that the insomnia group exhibited significantly reduced TST and lower sleep efficiency compared to the non-insomnia group both preoperatively and postoperatively (**Figures 3a, b**). Within-group analyses revealed no statistically significant changes in TST or SE from preoperative baseline in either group during the postoperative period (*P* > 0.05), indicating preserved sleep duration and efficiency relative to preoperative status. Furthermore, the insomnia group manifested prolonged sleep latency. Notably, SL in the insomnia group significantly increased on both the operative night (*P* = 0.018) and postoperative day1 (*P* = 0.022) compared to baseline, whereas no significant postoperative SL prolongation was observed in the non-insomnia group (**Figure 3c**). Sleeping characteristics analysis identified significant REM sleep duration reduction in the insomnia group specifically on the operative night (*P* = 0.031) and postoperative day1 (*P* = 0.037), with no significant alterations in NREM sleep stages (**Figures 3d, e**). Additionally, the insomnia group demonstrated higher microarousals across all timepoints. Longitudinal assessment revealed persistent microarousals elevation in the insomnia group from the operative night through postoperative day2 compared to baseline (all *P* < 0.05). In contrast, the non-insomnia group only exhibited transient microarousals increase on the operative night (*P* = 0.040) without sustained elevation thereafter (**Figure 3f**).

**Figure 4:**
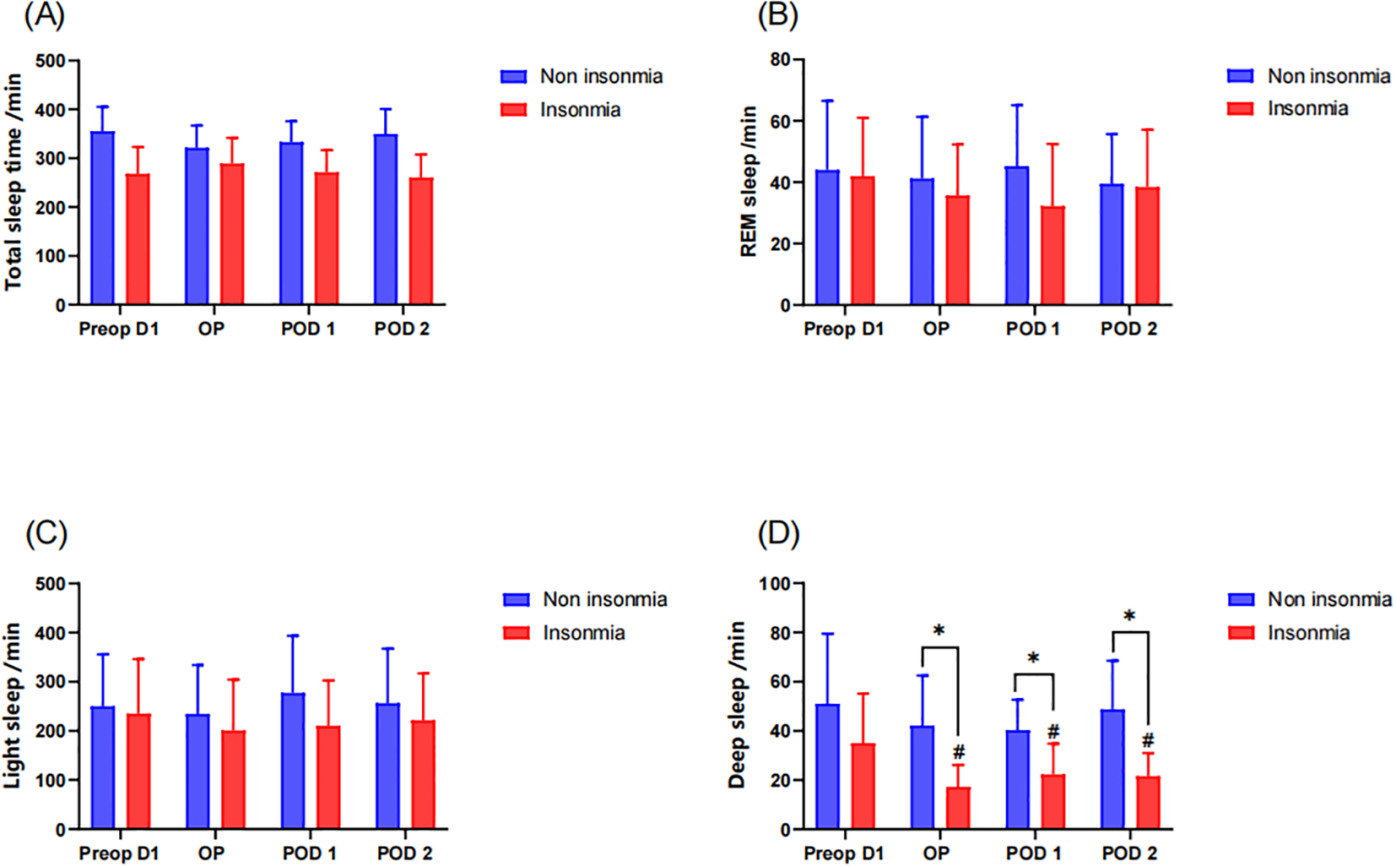
Postoperative sleep assessment by sleep tracking wristband: * indicates a significant difference between the insomnia group and the non-insomnia group, while the # represents a significant difference compared to pre-operation D1. **(A)** Total sleep time; **(B)** REM sleep; **(C)** light sleep; **(D)** deep sleep.

Objective sleep assessment via sleep tracking wristband further revealed no significant intergroup differences in TST, REM sleep duration, and light sleep duration (**Figures 4a–c**). While preoperative deep sleep duration showed comparable levels between groups, longitudinal assessment demonstrated that the insomnia group exhibited significant reductions in deep sleep from the operative day to postoperative day2 compared to preoperative baseline. Moreover, the insomnia group maintained significantly lower deep sleep than the non-insomnia group during these postoperative periods (**Figure 4d**).

## 4 Discussion

Sleep is a vital component of well-being. Postoperative sleep disturbance represents an important research field as sleep disturbance in the early postoperative period has a negative impact on health-related quality of life and can adversely affect the outcomes of surgery (3, 23). This prospective cohort study yields several pivotal insights regarding preoperative insomnia in elderly arthroplasty patients. Pre-existing insomnia exacerbates postoperative sleep fragmentation, manifested by persistently elevated microarousals (operative night to POD2) and prolonged sleep latency. Selective REM suppression occurs in insomnia patients during the acute postoperative phase (operative night and POD1), independent of NREM duration alterations. These findings to some extent substantiate the hypothesis that preoperative insomnia establishes a neurobiological vulnerability to postoperative sleep disruption.

There have been many studies on the sleep disorders after TKA or THA (25, 26). Previous studies have demonstrated the impact of general anesthesia on postoperative sleep quality (21, 27). This article mainly focuses on the changes in sleep quality of patients with preoperative insomnia after receiving spinal anesthesia. Some studies have found that patients experience a transient period of worsened sleep in the immediate postoperative period, whereas others have found no such evidence (12, 26). Our results demonstrate that patients with preoperative insomnia comorbidity exhibited significantly higher ISI and AIS scores throughout the perioperative period compared to non-insomnia controls. Crucially, neither group showed statistically significant changes in these subjective scores postoperatively versus preoperative baselines, indicating stable self-reported sleep quality after surgery. However, objective monitoring via EEG-based sleep monitor revealed exacerbated sleep fragmentation in the insomnia group, characterized by prolonged persistence of elevated microarousals and REM suppression. A prospective observational study monitored ambulatory polysomnography in 10 patients undergoing total hip or knee arthroplasty from the night before surgery to the fourth night after surgery, finding severe disruption of sleep structure on the first postoperative night, characterized mainly by the almost complete absence of rapid eye movement sleep and increasing awake time, which recovered to normal levels by the fourth night after surgery (16). Our study yielded similar results in the insomnia group, while the non-insomnia group did not exhibit such differences. The elevation in microarousals observed in the insomnia group represents a state of sustained sleep instability. This hyperarousal phenotype is increasingly recognized as a key substrate for daytime functional impairment and neurocognitive deficits. REM sleep is implicated in emotional regulation, memory consolidation and neuronal repair. The selective suppression of REM sleep during the postoperative phase may therefore contribute to the higher incidence of postoperative cognitive dysfunction and delirium observed in vulnerable populations. Further studies are needed to directly confirm and link these specific sleep metrics to functional outcomes.

The relationship between pain and sleep is bidirectional (28, 29). Pain can disrupt sleep, while sleep disturbance can lower pain thresholds (17, 30). A meta-analysis with 1226 participants showed that clinically significant preoperative insomnia symptoms were associated with moderate to severe pain intensity on the first postoperative day (31). However, in our cohort, patients with preoperative insomnia did not demonstrate elevated postoperative pain intensity compared to non-insomnia controls, as evidenced by statistically equivalent visual analog scale (VAS) scores. In this study, patients received standardized multimodal analgesia, achieving effective postoperative pain control throughout the assessment period. This outcome demonstrates that sleep quality around the time of surgery appears to be multifactorial, affected not only by the pain control (32, 33).

Polysomnography (PSG) remains the gold standard for sleep monitoring, providing comprehensive details on sleep architecture and physiological parameters (34). However, PSG is a relatively expensive and complex tool requiring dedicated sleep laboratories, specialized technicians for manual interpretation, and multiple electrode attachments that cause patient discomfort and restricted mobility. In recent years, wearable devices have emerged as promising alternatives to PSG for sleep assessment (35, 36). The EEG-based monitor used in this study is a single-channel wearable device affixed to the forehead that wirelessly transmits EEG signals for automated analysis. Previous validation studies have established strong concordance between the device and PSG in diagnosing sleep stages and monitoring sleep parameters, with reported sensitivity of 83.17% and specificity of 96.15% (24). Consequently, we used this validated device to assess perioperative sleep patterns in elderly patients undergoing THA/TKA, ensuring reliable data collection that provides clinically actionable insights for perioperative sleep management.

This study employed a comprehensive sleep evaluation protocol integrating. Three validated subjective instruments (PSQI, AIS, ISI) were used to evaluate multidimensional insomnia phenotyping. Objective verification via EEG sleep monitor and sleep tracking wristband captured complementary aspects of sleep structure. This approach exceeds the methodological rigor of prior studies relying solely on questionnaires. The implementation of standardized multimodal analgesia achieved equivalent pain control between groups. This design feature isolates the independent effect of preoperative insomnia on postoperative sleep disruption, overcoming a key limitation in observational pain-sleep research. As a study focusing exclusively on elderly arthroplasty patients, our findings provide additional evidence in this vulnerable population.

There also are potential limitations to this study. This study implemented a multimodal sleep assessment protocol, revealing discrepancies between measurement modalities. As the gold standard for sleep monitoring, polysomnography (PSG) was not performed due to its operational complexity and time-intensive nature, representing a methodological limitation. However, Sleep monitoring was performed using EEG-based sleep monitor in our study, enabling relatively objective and precise assessment of sleep architecture and quality parameters. Furthermore, the study focused exclusively on sleep disturbances during the acute postoperative phase, lacking longitudinal assessment of long-term sleep recovery (e.g., at 12-month follow-up). The underlying relationship and mechanism between the preoperative existing insomnia and postoperative sleep disturbances still requires further exploration.

## 5 Conclusion

This prospective cohort study establishes that preoperative insomnia independently exacerbates postoperative sleep fragmentation in elderly arthroplasty patients, manifested by sustained elevation of microarousals, prolonged sleep latency, selective REM suppression. And the study highlights the significance of perioperative sleep management for elderly patients. However, further studies are needed to validate the robustness of our findings and explain the mechanism.

## Data Availability

The raw data supporting the conclusions of this article will be made available by the authors, without undue reservation.
